# [Retracted] Activating autophagy and ferroptosis of 3‑Chloropropane‑1,2‑diol induces injury of human umbilical vein endothelial cells via AMPK/mTOR/ULK1

**DOI:** 10.3892/mmr.2024.13377

**Published:** 2024-10-29

**Authors:** Xin Yi, Xiao Long, Canzhang Liu

Mol Med Rep 27: 76, 2023; DOI: 10.3892/mmr.2023.12963

Following the publication of this paper, it was drawn to the Editor's attention by a concerned reader that the flow cytometric assay data shown in Fig. 2C on p. 5, certain of the fluorescence microscopy data shown in Fig. 4B on p. 7, the FerroOrange-stained cellular data in Fig. 5B and the C11-BODIPY581/591-stained cellular data in Fig. 5C on p. 8, and the cell autophagic data in Fig. 6E and G on p. 9 were strikingly similar to data that had already been submitted for publication in different form in different articles written by different authors at different research institutes (a few of which have now been retracted).

Owing to the fact that the contentious data in the above article had already been published, or were already under consideration for publication, prior to its submission to *Molecular Medicine Reports*, the Editor has decided that this paper should be retracted from the Journal. The authors were asked for an explanation to account for these concerns, but the Editorial Office did not receive a satisfactory reply. The Editor apologizes to the readership for any inconvenience caused.

